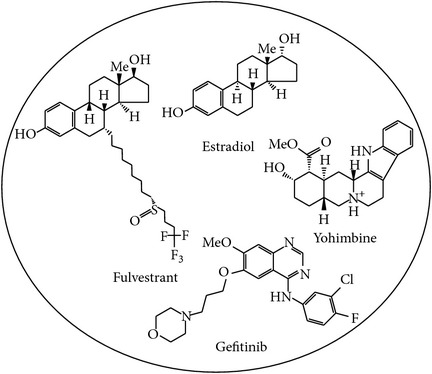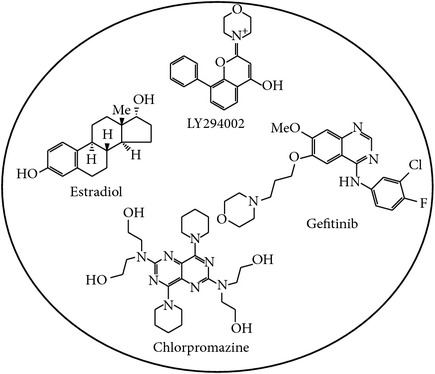# Network Biomarkers of Bladder Cancer Based on a Genome-Wide Genetic and Epigenetic Network Derived from Next-Generation Sequencing Data

**DOI:** 10.1155/2016/4149608

**Published:** 2016-02-29

**Authors:** Cheng-Wei Li, Bor-Sen Chen

**Affiliations:** Lab of Control and Systems Biology, National Tsing Hua University, Hsinchu 30013, Taiwan

## Abstract

Epigenetic and microRNA (miRNA) regulation are associated with carcinogenesis and the development of cancer. By using the available omics data, including those from next-generation sequencing (NGS), genome-wide methylation profiling, candidate integrated genetic and epigenetic network (IGEN) analysis, and drug response genome-wide microarray analysis, we constructed an IGEN system based on three coupling regression models that characterize protein-protein interaction networks (PPINs), gene regulatory networks (GRNs), miRNA regulatory networks (MRNs), and epigenetic regulatory networks (ERNs). By applying system identification method and principal genome-wide network projection (PGNP) to IGEN analysis, we identified the core network biomarkers to investigate bladder carcinogenic mechanisms and design multiple drug combinations for treating bladder cancer with minimal side-effects. The progression of DNA repair and cell proliferation in stage 1 bladder cancer ultimately results not only in the derepression of miR-200a and miR-200b but also in the regulation of the TNF pathway to metastasis-related genes or proteins, cell proliferation, and DNA repair in stage 4 bladder cancer. We designed a multiple drug combination comprising gefitinib, estradiol, yohimbine, and fulvestrant for treating stage 1 bladder cancer with minimal side-effects, and another multiple drug combination comprising gefitinib, estradiol, chlorpromazine, and LY294002 for treating stage 4 bladder cancer with minimal side-effects.

## 1. Introduction

Bladder cancer is still one of the most common cancers worldwide. Single gene markers have been proposed for improving cancer treatment [1]. However, single gene markers cannot overcome treatment side-effects because the markers are not implicated in genome-wide networks, and the analysis of a genome-wide network is a complicated issue from a systems biology perspective. The rapid development of molecular biology techniques has produced a great deal of high-throughput experimental data, including genome-wide microarray data, genome-wide methylation profiles, next-generation sequencing (NGS) data, microRNA (miRNA) profiles, genetic sequences, protein abundance data, and drug response genome-wide microarray data. These kinds of omics data provide an opportunity to design multiple drug combinations for the treatment of bladder cancer by applying the network biomarkers identified by systems biology.

To date, genetic regulation systems, including protein-protein interaction networks (PPINs) and gene regulatory networks (GRNs), have been applied to analyze the functional mechanisms behind human aging and cancer [2, 3]. We now know that epigenetic alterations are much more rapid and adaptive with regard to influencing genome-wide gene expression than genetic changes [4]. Rapid and slow response mechanisms, that is, epigenetic alterations and genetic changes, respectively, coordinate an efficient and robust system. Epigenetic regulation, including DNA methylation and histone modification, results in potentially reversible alterations in gene expression that do not involve permanent changes to the DNA sequence. miRNAs that are influenced by aberrant epigenetic regulation also mediate the regulation of gene expression [5]. It has been found that DNA methylation directly affects the binding affinities of miRNAs, RNA polymerase, and transcription factors (TFs) [6] and indirectly influences protein-protein interactions (PPIs) [7]. Methylation analysis of human genomic DNA in 12 tissues revealed that DNA methylation profiles are tissue-specific [8]. Therefore, omics data and systems biology methods [9–11] are required to unravel the mechanisms underlying carcinogenesis from the complex molecular biology and design anticancer drugs for the treatment of bladder cancer.

The Human Genome Project (HGP) has identified 30,000–40,000 genes in human DNA, including miRNAs. The genes, proteins, and their associations, miRNA regulation, and DNA methylation constitute the integrated genetic and epigenetic genome-wide network (IGEN), which coordinates cellular responses. PPIN in human lung cancer [12] and GRN in human aging [13] of the genes with significant expression differences between cancer cells (or aged people) and normal cells (or young people) have been identified for the extraction of the core network biomarkers according to the estimated association abilities between TFs (or upstream proteins) and target genes (or target proteins). Aging is associated with cancer [14]. The association abilities estimated by the network models assume that the binding affinities of TFs (or upstream proteins) to target genes (or proteins) are the same. According to a recent study in primary human somatic and germline cells [6], the impact of the binding affinities of miRNAs, RNA polymerase, and TFs on gene expression is mediated by DNA methylation. According to the available genome-wide methylation profiles and NGS data for bladder cancer in The Cancer Genome Atlas (TCGA), DNA methylation and miRNA regulation can be also characterized by the GRN model to identify the genome-wide IGEN. In this study, we identified the IGENs in normal bladder cells and bladder cancer cells and then investigated the impact of epigenetic regulation and miRNA regulation on bladder carcinogenesis by comparing the IGEN in normal bladder cells with that in bladder cancer cells.

Although a genome-wide IGEN can be identified based on well-defined system identification techniques [2, 12], the mean by which the core network biomarkers are extracted from the identified genome-wide network is still an important issue. The total association capabilities of a single node can affect the contribution it makes to its neighbors. However, the genome-wide IGEN including transcriptional gene regulations, miRNA regulations, and PPIs constitutes a genome-wide network structure. The contribution made by one node to its neighbors is not sufficient to explain its impact on a genome-wide scale network of bladder cells. In this study, we applied a principal genome-wide network projection (PGNP) based on principal component analysis (PCA) to identify core network biomarkers in bladder carcinogenesis, with the objective of extracting the most significant part from a genome-wide network structure. Because the drug response genome-wide microarray data are now available [15], we analyzed the drug response microarray data of the core network biomarkers to design multiple drug combinations with minimal side-effects for bladder cancer treatment. Therefore, the identified core network biomarkers could provide an opportunity to design such drug combinations for bladder cancer treatment. Furthermore, it has been reported that aging (over 45 years old) and smoking are two major risk factors for bladder carcinogenesis [16]. Therefore, we used the core network biomarkers to elucidate the cellular mechanisms by which aging and smoking elevate bladder cancer risk through epigenetic regulation, miRNA regulation, and signaling pathways.

**Figure 1:**
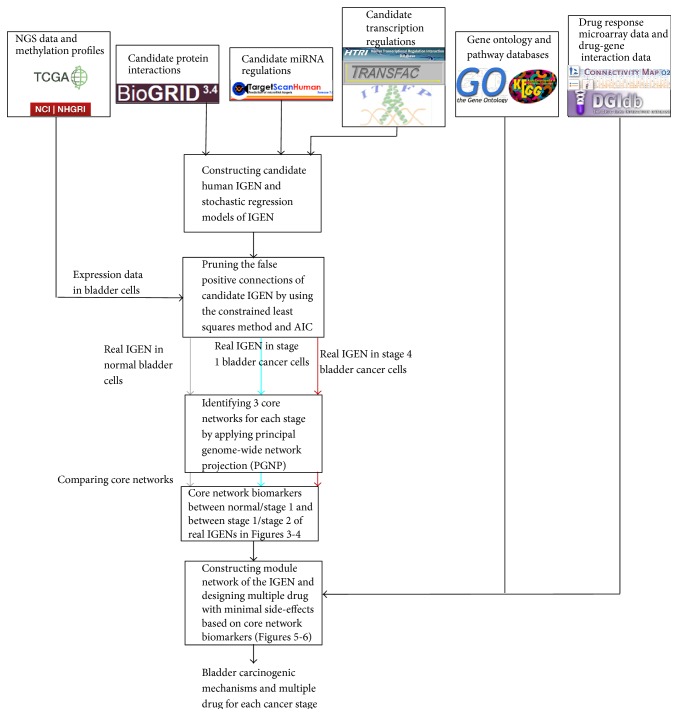
Flowchart of the proposed method for constructing the core network biomarkers and identifying bladder carcinogenesis mechanisms.

According to the strategy shown in the flowchart (Figure 1), we integrated omics data, including genome-wide methylation profiles, NGS expression data, miRNA profiles in TCGA, drug response genome-wide microarray data in the Connectivity Map (CMAP) [15], drug-gene interaction data in the Drug Gene Interaction Database (DGIdb) [17], miRNA-target gene association data in TargetScan [18], PPIs in BioGRID, transcription regulations in the Human Transcriptional Regulation Interactions database (HTRIdb) [19], the Integrated Transcription Factor Platform (ITFP) [20], and the TRANSFAC [21], biological processes and pathways in a gene ontology (GO) database, the National Center for Biotechnology Information (NCBI) Entrez Gene database, and the Kyoto Encyclopedia of Genes and Genomes (KEGG) pathway database [22]. We used miRNA-target gene association data, PPIs, and transcription regulations to build the candidate IGEN for general molecular mechanisms. We then constructed a regression IGEN model to characterize the molecular mechanisms including miRNA regulation, PPIs, transcription regulation, and DNA methylation in cells. To prune the false positive connections in the candidate IGEN and identify the model parameters of the IGEN in the real human bladder cells, we used methylation profiles, NGS expression data, and miRNA profiles in normal bladder cells and stage 1 and stage 4 bladder cancer cells. We then applied the constrained least squares method and the Akaike information criterion (AIC) [23], a system order detection method, to prune the false positive connections for obtaining the real IGENs in the three stages of human bladder carcinogenesis. The three genome-wide real IGENs in normal bladder cells and stage 1 and stage 4 bladder cancer cells were then projected into the three core networks of the three stages of bladder carcinogenesis, respectively. Because the core networks contain the identified signal transduction pathways, that is, the receptors and TFs of the core network can be directly or indirectly connected by the core proteins/TFs, the proteins/TFs, and the corresponding genes that participate in the identified signaling pathways of the core networks are considered as the core network biomarkers for normal and cancerous cells, respectively. The miRNAs with very different connections in regulating the genes of the core network biomarkers between two cells are also involved in the core network biomarkers. By comparing the identified connections of the IGENs, we investigated how the connection changes of the core network biomarkers from normal bladder cells to stage 1 bladder cancer cells and from stage 1 bladder cancer cells to stage 4 bladder cancer cells contribute to bladder carcinogenesis.

We also investigated how the module network of the core network biomarkers, including the KEGG pathways and biological processes, participates in bladder carcinogenesis. According to the information on the biological processes and signaling pathways in the GO database, the NCBI Entrez Gene database, and the KEGG pathway database, the roles of the TFs/proteins in the core network biomarkers are projected into three pathways: the SUMOylation, ubiquitination, and proteasome (SUP) pathway; the tumor necrosis factor (TNF) signaling pathway; and the endoplasmic reticulum (ER) signaling pathway. The roles of the downstream genes in the core network biomarkers are projected into three biological processes: cell proliferation, DNA repair, and metastasis. The module network, including the KEGG pathways, TFs, miRNAs, and biological processes, is connected according to the three identified IGENs in the three types of bladder cell. By comparing the connection changes of the module networks from normal bladder cells to stage 1 bladder cancer cells, and from stage 1 bladder cancer cells to stage 4 bladder cancer cells, we ultimately unraveled the cellular mechanisms behind bladder carcinogenesis and proposed two multiple drug combinations for treating stage 1 and stage 4 bladder cancers, respectively.

Additionally, to determine how the two major risk factors, aging and smoking, influence bladder carcinogenesis, we highlighted not only the significantly expressed genes between smokers and nonsmokers, but also the significantly expressed genes between young (≤45 years old) and old (>45 years old) people in the core network biomarkers of bladder carcinogenesis. Finally, we investigated the carcinogenic mechanism of human bladder cells by which the identified major factors, including downregulated miR-1-2, aging, and smoking, lead to the progression from normal bladder cells to stage 1 bladder cancer cells through the SUP and ER signaling pathways. The smoking-related protein HSP90AA1 and DNA methylation of* ECT2* mediate the progression from stage 1 bladder cancer cells to metastasis in stage 4 bladder cancer. Activated DNA repair and accumulated epigenetic alterations lead to the phenotypic changes of bladder cells from normal to cancerous, and from cancerous to metastatic cells owing to the immortality of cancer cells. Based on the core network biomarkers in bladder carcinogenesis, a multiple drug combination comprising gefitinib, estradiol, yohimbine, and fulvestrant was designed for treating stage 1 bladder cancer with minimal side-effects, while a multiple drug combination comprising gefitinib, estradiol, chlorpromazine, and LY294002 was designed for treating stage 4 bladder cancer with minimal side-effects.

## 2. Materials and Methods

According to the flowchart in Figure 1, we constructed a candidate human IGEN by mining large databases, including BioGRID, TargetScan, HTRIdb, TRANSFAC, and ITFP. However, many false positive and insignificant connections existed in the candidate human IGEN for normal and cancerous bladder cells. Using the NGS expression data, miRNA profiles, and the methylation profiles of normal and cancerous bladder cells in TCGA, we identified the association parameters of the network connections. We also applied AIC to detect the systems order, that is, the number of connections, and to delete the insignificant connections that were out of system order to prune the false positive connections in the candidate IGEN and obtain the two real IGENs for normal and cancerous bladder cells, respectively. By applying PGNP to the two real IGENs in normal and cancerous cells, we first identified the core proteins/TFs that played a major role in the principal networks of the IGENs, constituting the core IGENs in normal and cancerous cells. To determine how the signaling cascades from the core receptor proteins to the core TFs participate in bladder carcinogenesis, the core proteins, which mediate the signal transductions from the core receptor proteins to the core TFs, and their corresponding genes were considered the core network biomarkers of the normal and cancerous cells. The miRNAs with very different connections in regulating the genes of the core network biomarkers between normal and cancerous cells were also involved in the core network biomarkers. Finally, by comparing the connection changes of the core network biomarkers from normal cells to stage 1 cancer cells, and from stage 1 cancer cells to stage 2 cancer cells, we investigated the cellular mechanisms of bladder carcinogenesis.

### 2.1. Data Preprocessing of Omics Data

We downloaded the genome-wide mRNA and miRNA NGS data and the methylation profiles from TCGA, including 17 samples for normal bladder cells, 348 samples for stage 1 bladder cancer cells, and 56 samples for stage 4, that is, metastatic stage, bladder cancer cells. The data also contained 6 samples for young (≤45 years old) people, 477 samples for old (>45 years old) people, 98 samples for nonsmokers, and 323 samples for smokers. We used one-way analysis of variance (ANOVA) to identify significant differences in gene expression between smokers and nonsmokers, and between young and old people (*p* value < 0.05). We used the gene symbols of the human gene information data downloaded from the NCBI FTP site as standard human gene names to integrate the omics data, including NGS data, methylation profiles, drug response genome-wide microarray data in CMAP, drug-gene interaction DGIdb data, miRNA-target gene association data in TargetScan, PPIs in BioGRID, transcription regulations in HTRIdb, and ITFP and TRANSFAC data. We also used the GO database, the NCBI Entrez Gene database, and the KEGG pathway database to find the biological processes and pathways of each gene. We used Matlab's text-file and string manipulation tools for text mining.

### 2.2. Construction of the Stochastic Regression Models for the IGEN System

The goal of the stochastic regression model is to characterize molecular mechanisms, including PPIs, transcription regulations, miRNA regulations, and epigenetic regulations via DNA methylation, by NGS data through detecting false positives of candidate IGENs in human cells. For the stochastic regression model of the gene regulatory subnetwork in the candidate human IGEN, including transcription regulations, miRNA regulations, and epigenetic regulations via DNA methylation, we identified the regulation capabilities of TFs and miRNAs in the GRN of the candidate IGEN. For the expression levels of the *i*th gene, its DNA methylation and its *j*th TF/protein and *l*th miRNA in the *n*th sample are denoted by *x*_*i*_(*n*), *m*_*i*_(*n*), *y*_*j*_(*n*), and *s*_*l*_(*n*), respectively. Then, the stochastic regression model of GRN is described by the following stochastic regression equation:

(1) xin=∑j≡Ωij≠iaijMinyjn+∑l≡δicliMinxinsln+biMin+νin,for  i=1,…,K,  n=1,…,N,

where the repression ability from the *l*th miRNA to the *i*th gene *c*_*li*_ ≤ 0; the basal level of the *i*th gene expression *b*_*i*_ ≥ 0; Ω_*i*_ ⊂ Ω ≡ {1, …, *K*}; *δ*_*i*_ ⊂ *δ* ≡ {1, …, *L*};  *M*_*i*_(*n*) = 1/[1 + (*m*_*i*_(*n*)/0.5)^2^]; Ω_*i*_ and *δ*_*i*_ denote the candidate regulations based on the databases of transcription regulation and miRNA-target association, respectively; *a*_*ij*_ indicates the regulatory ability from the *j*th TF *y*_*j*_(*n*) to the *i*th gene; *v*_*i*_(*n*) represents the stochastic noise due to the modeling residue and fluctuation in the *i*th gene; and *K*,  *L*, and *N* are the total number of TFs, miRNAs, and data samples in the omics data, respectively. *M*_*i*_(*n*) denotes the effect of methylation *m*_*i*_(*n*) on the binding affinity of TFs, miRNAs, or RNA polymerase on the *i*th gene which also represents the impact of DNA methylation of the *i*th gene on the binding affinities of miRNAs, RNA polymerase, and TFs in the gene expression process. The effect on binding affinities *M*_*i*_(*n*), for *i* = 1,…, *K*, ranged between 0.2 and 1, while the expression range of the genome-wide DNA methylation *m*_*i*_(*n*), for *i* = 1,…, *K*, is between 0 and 1. If DNA methylation of the *i*th gene is close to 1, the effect on the binding affinity to the *i*th gene is close to 0.2, which implicates the impact of DNA methylation on the binding affinities of miRNAs, RNA polymerase, and TFs to be like an inhibitor. The *i*th mRNA expression results from transcription regulations ∑_*j*≡*Ω*_*i*_,*j*≠*i*_ *a*_*ij*_ *M*_*i*_(*n*)*y*_*j*_(*n*), miRNA repressions ∑_*l*≡*δ*_*i*__ *c*_*li*_ *M*_*i*_(*n*)*x*_*i*_(*n*)*s*_*l*_(*n*), the mRNA basal expression *b*_*i*_ *M*_*i*_(*n*), and the stochastic noise due to measurement and random fluctuations *ν*_*i*_(*n*). In model (1), the TF regulations, miRNA regulations, and basal levels are all influenced by the DNA methylation *m*_*i*_(*n*) on the *i*th gene.

For the stochastic regression model of the miRNA regulatory subnetwork in the candidate IGEN, the expression levels of the *l*th miRNA and its *i*th target gene in the *n*th sample, denoted by *s*_*l*_(*n*) and *x*_*i*_(*n*), respectively, could be described by the stochastic regression model of miRNA regulatory network (MRN) as the following stochastic regression equation:

(2) sln=∑i≡δlcliMinxinsln+Mlnzl+eln,for  l=1,…,L,  n=1,…,N,

where the repression ability of the *l*th miRNA to the *i*th gene *c*_*li*_ ≤ 0; the basal level of the *l*th miRNA expression *z*_*l*_ ≥ 0; *δ*_*l*_ ⊂ *δ* ≡ {1, …, *L*}; *δ*_*l*_ denotes the candidate regulations of the *l*th miRNA based on the database of miRNA-target gene association; *e*_*l*_(*n*) represents the stochastic noise owing to the modeling residue and fluctuation in the *l*th miRNA. The *l*th miRNA expression in (2) results from miRNA-gene interactions ∑_*i*≡*δ*_*l*__ *c*_*li*_ *M*_*i*_(*n*)*x*_*i*_(*n*)*s*_*l*_(*n*), the miRNA basal expression *z*_*l*_, and the stochastic noise *e*_*l*_(*n*).

For the stochastic regression model of the PPI subnetwork in the candidate IGEN, the expression level of the *j*th protein and its *k*th connecting protein in *n*th sample, denoted by *y*_*j*_(*n*) and *y*_*k*_(*n*), respectively, could be described by the stochastic regression model of PPIN as the following stochastic regression equation:

(3) yjn=∑k≡Ωjk≠jdjkyknyjn+hj+wjn,for  j=1,…,K,  n=1,…,N,

where the basal level of the *j*th protein expression *h*_*j*_ ≥ 0; Ω_*j*_ ⊂ Ω ≡ {1,…, *K*}; Ω_*j*_ denotes the candidate interactions of the *j*th protein based on the PPI database; *d*_*jk*_ indicates the interaction ability of the *k*th protein to the *j*th protein; and *w*_*j*_(*n*) represents the stochastic noise owing to the modeling residue and fluctuation in the *j*th protein. The *j*th protein expression in (3) results from the rate of formation of the protein complex *y*_*k*_(*n*)*y*_*j*_(*n*) proportional to the product of the concentration of each protein ∑_*k*≡*Ω*_*j*_,*k*≠*j*_ *d*_*jk*_ *y*_*k*_(*n*)*y*_*j*_(*n*) [24, 25], the protein basal expression *h*_*j*_, and the stochastic noise *w*_*j*_(*n*).

We proposed general stochastic regression models to characterize cellular mechanisms, including genetic and epigenetic regulations, in human cells. A number of parameters, including the TF regulatory ability *a*_*ij*_, the miRNA repression ability *c*_*li*_, and the protein interaction ability *d*_*jk*_, needed to be estimated and were determined using the databases of PPI, miRNA-target gene association, and transcription regulation.

### 2.3. Identification of the TF Regulatory Ability *a*_*ij*_, the miRNA Repression Ability *c*_*li*_, and the Protein Interaction Ability *d*_*jk*_ and Their Statistical Significance Testing

We used the mRNA and miRNA expression data from the NGS as the expression levels for *x*_*i*_(*n*) and *s*_*l*_(*n*), respectively, and used DNA methylation profiles as the expression level of *m*_*i*_(*n*) to identify the model parameters *a*_*ij*_, *c*_*li*_, *d*_*jk*_, *b*_*i*_, *z*_*l*_, and *h*_*j*_ in (1)–(3). Because large-scale measurement of protein activities has yet to be realized and 73% of the variance in protein abundance can be explained by mRNA abundance [26], mRNA expression profiles were always used to substitute for the protein expression profiles. Therefore, we also applied mRNA expression levels in the NGS data as the expression levels of *y*_*j*_(*n*) to identify the parameters in (1)–(3). If the simultaneously measured genome-wide protein expression data and the mRNA expression data in each bladder cancer stage are available, the general models in (1)–(3) can also be applied to identify the real IGEN of the cancer more precisely. Because the parameters in (1) have certain constraints, such as the nonpositive miRNA repressions and nonnegative basal levels, the regulatory parameters were identified by solving the constrained least square parameter estimation problem in the following.

In order to identify the parameters in (1), the stochastic regression model of GRN was rewritten as the following linear regression form:

(4) xin=Miny1n⋯MinxinyKnMinxins1n⋯MinxinsLnMin,ai1⋮aiKc1i⋮cLibi+νin=ϕinθi1+νin,for  i=1,…,K,  n=1,…,N,

where *ϕ*_*i*_(*n*) denotes the regression vector and *θ*_*i*_^1^ is the parameter vector of target gene *i* to be estimated. *x*_*i*_(*n*) and *ϕ*_*i*_(*n*) are available in the omics data.

The regression model (4) at different data samples can be rearranged as follows:

(5) $$\begin{array}{c} \left[\begin{array}{c} x_{i}\left(1\right)\\ \vdots \\ x_{i}\left(N\right) \end{array}\right]=\left[\begin{array}{c} \phi_{i}\left(1\right)\\ \vdots \\ \phi_{i}\left(N\right) \end{array}\right]\theta_{i}^{1}+\left[\begin{array}{c} v_{i}\left(1\right)\\ \vdots \\ v_{i}\left(N\right) \end{array}\right], \end{array}$$

where *N* denotes the number of data samples in the NGS data of a bladder cancer stage.

For simplicity, we define the notations *X*_*i*_,  Φ_*i*_, and *V*_*i*_ to represent (5) as follows:

(6) $$\begin{array}{c} X_{i}=\Phi_{i}\theta_{i}^{1}+V_{i}. \end{array}$$

The constrained least square parameter estimation problem of *θ*_*i*_^1^ is formulated as follows:

(7) minθi1 Φiθi1−Xi22subject to 0⋯⋯0⋮⋱⋱⋮⋮⋱⋱⋮0⋯⋯0︷K 10⋯00⋱⋱⋮⋮⋱100⋯0−1︷L+1θi1≤0⋮⋮0.

This gives the constraints to force the miRNA repression *c*_*li*_ to be always nonpositive and the basal level *b*_*i*_ to be always nonnegative in (1); that is, *c*_*li*_ ≤ 0 and *b*_*i*_ ≥ 0. The constrained least square problem was solved using the active set method for quadratic programming [27, 28].

Similarly, the stochastic regression model of the miRNA regulatory subnetwork in (2) was rewritten in the following regression form:

(8) sln=M1nx1nsln⋯MKnxKnslnMlncl1⋮clKzl+eln=ϑlnθl2+eln,for  i=1,…,K,  n=1,…,N,

where *ϑ*_*l*_(*t*) indicates the regression vector and *θ*_*l*_^2^ is the parameter vector to be estimated.

For simplicity, we define the notations *S*_*l*_, Ψ_*l*_, and *E*_*l*_ to represent (8) as follows:

(9) $$\begin{array}{c} S_{l}=\Psi_{l}\theta_{l}^{2}+E_{l}. \end{array}$$

The parameter identification problem is then formulated as follows:

(10) minθi2 Ψlθl2−Sl22subject to 10⋯0⋱⋱⋮⋱10⋯0︷K0⋮0−1θl2≤0⋮⋮0.

This gives the constraint to force the miRNA repression *c*_*li*_ to be always nonpositive and the basal level *z*_*l*_ to be always nonnegative in (2); that is, *c*_*li*_ ≤ 0, and *z*_*l*_ ≥ 0. Finally, the protein model (3) uses the same way like above to make sure *h*_*j*_ ≥ 0.

**Figure 2:**
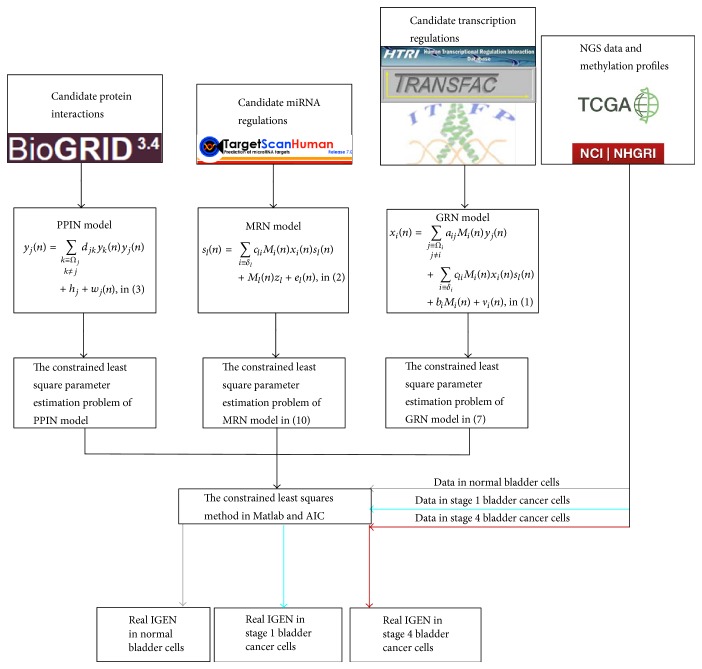
Flowchart of the proposed methodology to identify the IGENs for normal bladder cells, and stage 1 and 4 bladder cancer cells.

Furthermore, in order to extract the core network biomarkers from normal and cancerous cells, we first used NGS data and methylation profiles in the normal and stage 1 and 4 bladder cancer cells to identify an IGEN for normal bladder cells and a general IGEN for bladder cancer cells. The two identified IGENs were used to extract the core network biomarkers in bladder carcinogenesis. We then used the association parameters in the general IGEN of bladder cancer cells as the initial condition of the constrained least square parameter estimation and applied the data on stage 1 and 4 bladder cancer cells to identify the IGENs for stages 1 and stage 4, respectively. According to the three identified IGENs in normal bladder cells, and the stage 1 and 4 bladder cancer cells, we determined the cellular mechanisms of the core network biomarkers in bladder carcinogenesis. The proposed methodology to identify the IGENs for normal bladder cells and stage 1 and 4 bladder cancer cells was summarized in the flowchart in Figure 2.

By applying Student's *t*-test to the parameter estimation method [29], the *p* values for the estimated parameters, including the TF regulatory ability *a*_*ij*_, the miRNA repression ability *c*_*li*_, and the protein interaction ability *d*_*jk*_, were calculated to determine the significance of the parameters. Additionally, to determine the significance of expression level and DNA methylation profile of a gene/miRNA between normal bladder cells and cancerous bladder cells, we applied one-way ANOVA to calculate the *p* value.

After the parameter identification problem had been solved, we identified the IGEN for each bladder cell type. For example, we identified the regulatory parameter *a*_RPS20,JUN_ = 0.26 from the TF JUN to the target gene RPS20 (*p* value < 0.02) in stage 4 bladder cancer cells, the interaction parameter *d*_HUWE1,ADRM1_ = 1.2 between the two proteins ADRM1 and HUWE1 (*p* value < 0.005) in stage 1 bladder cancer cells, and the coupling rate *c*_RPS20,MIR155_ = −1.2 between the miRNA miR155 and the mRNA RPS20 in stage 4 bladder cancer cells (*p* value < 0.07).

### 2.4. Principal Genome-Wide Network Projection (PGNP)

After the identification of the IGENs in normal and cancer cells, we extracted the core network biomarkers of the IGENs based on the perspectives of the functional modules and pathways to reveal the cellular mechanisms behind bladder carcinogenesis. To extract the core network biomarkers, including the core proteins, their corresponding genes, and their upstream miRNAs, from an IGEN on a genome-wide scale, we first decomposed the combined network matrix of the IGEN to left- and right-singular vectors and singular values based on singular value decomposition (SVD). The top left- and right-singular vectors with the top singular values constitute the principal network of the IGEN. The projection distance of each gene/protein/miRNA to these top singular vectors represents the significance of this gene/protein/miRNA in the IGEN. The genes/proteins/miRNAs with the top projection distance ultimately constitute the core network biomarkers of the IGEN. Let the combined network matrix of the TF regulatory ability *a*_*ij*_, the miRNA repression ability *c*_*li*_, and the protein interaction ability *d*_*jk*_ of the IGEN in (1)–(3) be represented by

(11) $$\begin{array}{c} A=\left[\begin{array}{ccc} a_{11} & \cdots & a_{1K}\\ \vdots & \ddots & \vdots \\ a_{K1} & \cdots & a_{KK}\\ c_{11} & \cdots & c_{1K}\\ \vdots & \ddots & \vdots \\ c_{L1} & \cdots & c_{LK}\\ d_{11} & \cdots & d_{1K}\\ \vdots & \ddots & \vdots \\ d_{K1} & \cdots & d_{KK} \end{array}\right]. \end{array}$$

By applying PGP, the matrix *A* is then be decomposed as follows:

(12) AUDVT=u1⋯uKd1000⋱000dKv1⋯vKT=∑i=1KuidiviT,

where *u*_*i*_, *v*_*i*_ ∈ *ℜ* ^*K*^ are the *i*th left- and right-singular vectors of *A*, respectively. The diagonal entries of *D* are the *K* singular values of *A* in descending order, *d*_1_ ≥ ⋯≥*d*_*K*_.

The eigenexpression fraction (*E*_*m*_) is defined as

(13) $$\begin{array}{c} E_{m}=\frac{d_{m}^{2}}{\sum_{m=1}^{K}d_{m}^{2}}. \end{array}$$

We choose the top *M* singular vectors of *V* such that ∑_*m*=1_^*M*^ *E*_*m*_ ≥ 0.85, with the minimal *M*, so that the top *M* principal components contain 85% of the IGEN from an energy point of view. The principal projections of *A* to the top *M* singular vectors of *V*, or similarities, are defined as follows:

(14) Sk,m=ak·vmT,for  k=1,…,2K+L,  m=1,…,M,

where *a*_*k*_ and *v*_*m*_^*T*^ denote the *k*th row vector of *A* and the *m*th singular vector of *V*, respectively. Furthermore, we defined the 2-norm distance from the target genes, miRNAs, and proteins/TFs to the top *M* singular vectors, respectively, as follows:

(15) Dk=∑m=1MSk,m21/2,for  k=1,…,2K+L,

where *D*(*k*) for *k* = 1,…, *K*, for *k* = *K* + 1,…, *K* + *L*, and for *k* = *K* + *L* + 1,…, 2*K* + *L* are the 2-norm distances from the target genes, miRNAs, and proteins/TFs to the top *M* singular vectors, respectively. According to *D*(*k*) for *k* = *K* + *L* + 1,…, 2*K* + *L*, we can identify the core proteins/TFs that play a major role in the principal networks of the IGENs, constituting the core IGENs in normal and cancer cells. The identified core proteins/TFs contain receptors that mediate the signaling cascades connected to core TFs. The core proteins, which participate in signal transduction from core receptors to core TFs, and their corresponding genes, were considered the core network biomarkers for normal and cancerous cells. The miRNAs with very different connections in regulating the genes of the core network biomarkers between two cells were also involved in the core network biomarkers.

### 2.5. Design of a Multiple Drug Combination with Minimal Side-Effects for the Treatment of Bladder Cancer

To design a multiple drug combination with minimal side-effects for the treatment of bladder cancer based on the core network biomarkers of the IGEN, we considered two databases, CMAP and DGIdb. CMAP contains the genome-wide microarray data in response to 1327 drugs in five cell lines, while DGIdb comprises a drug-gene interaction database. Multiple drug therapy induces a genome-wide response. The strategy of multiple drug screening is that the multiple drugs should inhibit the highly expressed genes, activate the reduced expression of the genes, and not influence the nondifferentially expressed genes in the core network biomarkers of bladder cancer cells compared with normal bladder cells. The binding protein of the designed multiple drug combination can also be obtained using the DGIdb. The strategy leads to improved drug safety and efficacy in the treatment of bladder cancer.

## 3. Results and Discussion

### 3.1. Construction of IGEN

**Figure 3:**
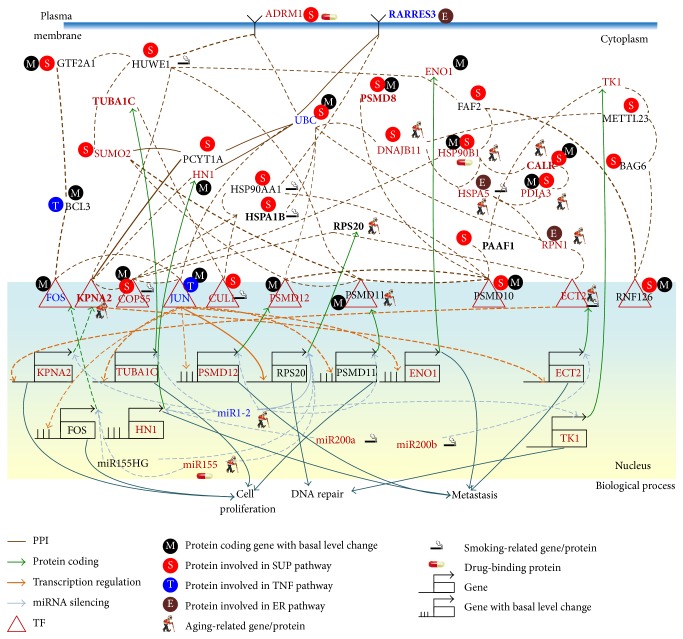
Comparison of genetic and epigenetic alterations and connection changes in the core network biomarkers of bladder carcinogenesis between normal bladder cells and stage 1 bladder cancer cells. Red, blue, and black gene/miRNA symbols represent the highly expressed genes, the suppressed genes, and the nondifferentially expressed genes in stage 1 bladder cancer cells, respectively, compared with normal bladder cells. Dashed and solid lines denote the identified connections in normal and cancerous cells, respectively. The identified connections of the core network biomarkers do not exist in normal bladder cells only. Bold lines indicate the high regulatory or interaction parameters, that is, *a*_*ij*_, *c*_*li*_, and *d*_*jk*_, identified in the stochastic regression models (1)–(3) of the IGEN. The bold proteins, including RARRES3, TUBA1C, PSMD8, HSPA1B, RPS20, CALR, PAAF1, and KPNA2, were the identified core network biomarkers. The major factors, including downregulated miR1-2, the aging-related proteins, HSP90B1, CALR, HSPA5, PDIA3, RPN1, and ECT2, the smoking-related proteins, HUWE1, HSPA5, and ECT2, and the epigenetic regulation of* ENO1*,* HSP90B1*,* CALR*, and* PDIA3*, lead to the progression from normal bladder cells to stage 1 bladder cancer cells through the SUP and ER signaling pathways.

**Figure 4:**
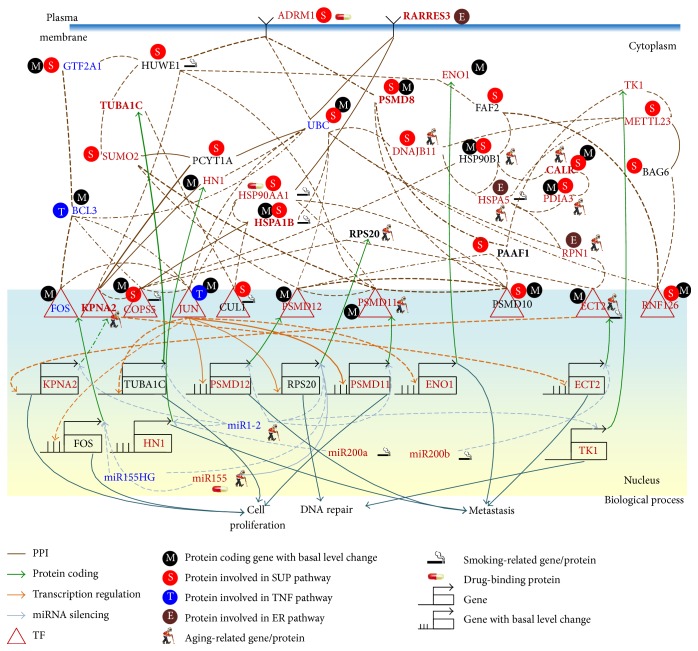
Comparison of genetic and epigenetic alterations and connection changes in the core network biomarkers of bladder carcinogenesis between stage 1 and stage 4 bladder cancer cells. Red, blue, and black gene/miRNA symbols represent the highly expressed genes, the suppressed genes, and the nondifferentially expressed genes in stage 4 bladder cancer cells, respectively, compared with stage 1 bladder cancer cells. Dashed, dash-dot, and solid lines denote the identified connections in stage 1 cancer cells, stage 4 cancer cells, and both stage 1 and 4 cancer cells, respectively. Bold lines indicate the high regulatory or interaction parameters, that is, *a*_*ij*_, *c*_*li*_, and *d*_*jk*_, identified in the stochastic regression models (1)–(3) of the IGEN. The bold proteins RARRES3, TUBA1C, PSMD8, HSPA1B, RPS20, CALR, PAAF1, and KPNA2 were the identified core network biomarkers. The smoking-related protein HSP90AA1 and DNA methylation of* ECT2* mediate metastasis of bladder cancer.

We first used NGS expression data and methylation profiles in normal bladder cells and stage 1 and 4 bladder cancer cells to identify a real IGEN for normal bladder cells and a general real IGEN for bladder cancer cells (see Section 2). By applying PGNP to the real IGEN of the normal bladder cells and the general real IGEN of the bladder cancer cells, we then obtained 115 core proteins/TFs for the core IGEN of the normal bladder cells and 138 core proteins/TFs for the core IGEN of the bladder cancer cells. To determine how the signaling cascades from the core receptor proteins to the core TFs participate in bladder carcinogenesis, the core proteins, which mediate the signal transductions from core receptor proteins to core TFs, and their corresponding genes were considered the core network biomarkers. The miRNAs with a high number of different connections regulating the genes of the core network biomarkers between normal and cancerous cells were also involved in the core network biomarkers. Moreover, to identify the mechanism of carcinogenesis from stage 1 to stage 4 bladder cancer, we used the identified parameters of models (1)–(3) in the general IGEN of bladder cancer cells as the initial condition of the constrained least square parameter estimation. We then applied the data for stage 1 and 4 bladder cancer cells to obtain the two real IGENs for stage 1 and 4 bladder cancer, respectively. Furthermore, we analyzed the connection changes of the core network biomarkers between normal bladder cells and stage 1 bladder cancer cells (Figure 3) and between stage 1 and 4 bladder cancer cells (Figure 4) to determine the mechanisms of bladder carcinogenesis and accordingly design multiple drug combinations for treating bladder cancer with minimal side-effects.

To investigate the impact of the major risk factors, aging and smoking, on the core network biomarkers of bladder carcinogenesis, we highlighted the significantly expressed genes between young and old people and between nonsmokers and smokers in the core network biomarkers (*p* value < 0.05). Additionally, the genes with changes in the basal level of (1) between normal bladder cells and stage 1 bladder cancer cells and between stage 1 and 4 bladder cancer cells were also highlighted in the core network biomarkers of Figures 3 and 4, respectively. The basal level change of a gene between two cell types has been implicated in the epigenetic regulation of gene expression. The expression of a gene that exhibits a basal level change and a significant change (*p* value < 0.05) of its methylation profile between the two bladder cell types is probably regulated by DNA methylation in bladder carcinogenesis.

### 3.2. Projection of the Core Network Biomarkers into Biological Processes and Signaling Pathways to Investigate Carcinogenic Mechanisms of Bladder Cancer

According to the information of the biological processes and signaling pathways in the GO and KEGG pathway databases, the roles of the genes in the core network biomarkers (Figures 3 and 4) are projected into three pathways: the SUP, TNF signaling, and ER signaling pathways and three biological processes: cell proliferation, DNA repair, and metastasis.

It has been reported that the SUP pathway is associated with increased proliferation in urinary bladder carcinogenesis [30]. HuaChanSu (HCS), a class of toxic steroids, has been used to show that the TNF pathway mediates the inhibition of cell proliferation in bladder cancer [31]. Moreover, the viability of human bladder cancer cells is reduced by using cantharidin, a natural toxin, through the ER pathway [32]. Therefore, the proteins of the core network biomarkers participating in the SUP, TNF, and ER signaling pathways play an important role in bladder carcinogenesis. We then determined how the core network biomarkers mediate bladder carcinogenesis through the influences of aging, smoking, epigenetic regulation, and miRNA regulation.

The role of the SUP pathway is to degrade misfolded proteins, influence PPIs, translocate proteins, and stabilize protein structure. Owing to the accumulation of genetic mutations and epigenetic alterations in cancer cells, the SUP pathway plays a crucial role in the maintenance of many important cellular processes in cancer cells. The repressed activity of ubiquitin C (UBC), which encodes the polyubiquitin precursor, influences degradation and translation of several proteins in stage 1 and stage 4 bladder cancer cells. For example, the repression of UBC affects the signal transduction of RARRES3, a tumor suppressor, in bladder carcinogenesis. To maintain the cellular functions of cancer cells, the regulation of the SUP pathway adapts to the accumulated genetic mutations and epigenetic alterations.

In normal cells, the TNF pathway is critical for inducing inflammation, which can cause cell death. Accumulated DNA damage, epigenetic alterations, or stresses can induce the TNF pathway, and the pathway then triggers cell death. JUN, one of the TFs in the TNF pathway, plays an important role in promoting the invasion and migration of bladder cancer cells [33]. We determined that the repressed expression of JUN in stage 1 bladder cancer cells leads to cancer cell immortality and causes accumulated genetic mutations and epigenetic alterations. Additionally, the results revealed that JUN was activated in stage 4 bladder cancer cells to mediate metastasis. The role of JUN in the metastasis of bladder cancer cells can also be supported [33]. It has also been reported that the TNF pathway acts as a switch between inflammation and cancer [34]. Moreover, downregulated BCL3, which participates in the TNF pathway in the adipose tissue of the bladder wall, leads to reduced inflammation in bladder carcinogenesis [35].

The ER pathway participates in the regulation of protein folding, protein synthesis, and posttranslational modifications [36]. Misfolded proteins, arising from genetic mutations, epigenetic alterations, or stresses, induce the ER pathway to restore cellular homeostasis in normal cells. Owing to the immortal nature of cancer cells, the accumulated genetic mutations and epigenetic alterations in bladder cancer cells can activate most of the genes that contribute to the ER pathway in bladder carcinogenesis (Figures 3 and 4). In the ER pathway, only RARRES3, a tumor suppressor gene, was downregulated in stage 1 bladder cancer cells.

### 3.3. The Impact of Aging, Smoking, and miRNA and Epigenetic Regulation on Bladder Carcinogenesis through the Core Network Biomarkers

Major factors, including downregulated miR1-2 and aging- and smoking-related proteins, may lead to the progression from normal bladder cells to stage 1 bladder cancer cells through the SUP and ER signaling pathways.

It has been reported that aging and smoking are the major factors that accumulate genetic and epigenetic alterations and ultimately induce bladder carcinogenesis. In Figure 3, our results reveal that ADRM1 regulates KPNA2, which promotes proliferation, and is mediated by the aging-related proteins, HSP90B1, CALR, HSPA5, PDIA3, RPN1, and ECT2, the smoking-related proteins, HUWE1, HSPA5, and ECT2, and the epigenetic regulation of ENO1, HSP90B1, CALR, and PDIA3, through the SUP and ER signaling pathways. ADRM1 knockdown leads to a reduction of cancer cell proliferation and has been found in gastric [37], ovarian [38], liver [39], and colorectal cancers [40] and acute leukemia [41]. Therefore, the results support the hypothesis that aging is the most important factor in inducing bladder carcinogenesis through the SUP pathway. Additionally, our results (Figure 3) show that the inhibited aging-related miRNA miR1-2 in stage 1 bladder cancer cells leads to miR1-2 dysregulation of genes including* KPNA2*,* TUBA1C*,* HN1*,* PSMD11*,* PSMD12*, and* TK1*, which influence cell proliferation, DNA repair, and metastasis. miR1-2 has also been identified as a tumor suppressor in bladder cancer cells [42].

### 3.4. miR1-2 and miR200b Mediate the Reduction of Cell Proliferation and Metastasis through KPNA2 and ECT2, Respectively

The receptor ADRM1 signal triggers the signaling cascade from the smoking-related protein HUWE1 to the aging-related proteins HSP90B1 and RPS20 and the smoking-related TF COPS5. The TF COPS5 upregulates the metastasis-associated gene* ECT2*, which is suppressed by miR200b in stage 1 bladder cancer cells. The results show the cross-regulation between the transcription of the smoking-related protein COPS5 and the aging-related protein ECT2. The aging-related miRNA miR1-2 and the smoking-related miRNA miR200b act as a switch to depress the proliferation-associated protein KPNA2 in stage 1 and stage 4 bladder cancer cells (Figures 3 and 4) and the metastasis-associated gene* ECT2* in stage 4 bladder cancer cells (Figure 4), respectively.

### 3.5. The Smoking-Related Protein HSP90AA1 and DNA Methylation of ECT2 Mediate the Metastasis of Bladder Cancer

Our results reveal that receptor RARRES3 signaling triggers the activated TF JUN mediated by the smoking-related protein HSP90AA1, and JUN then activates the metastasis-associated gene* PSMD12* in stage 4 bladder cancer cells (Figure 4). Receptor ADRM1 signaling also triggers the metastasis-associated protein PSMD12 through the proteins PSMD8 and PAAF1 and epigenetic regulation in stage 4 bladder cancer cells. This shows that metastasis-associated* ECT2* is activated by epigenetic regulation in stage 4 bladder cancer cells. Receptor RARRES3 signaling also triggers the aging-related and proliferation-associated TF PSMD11 through the smoking-related protein HSP90AA1 in stage 4 bladder cancer cells. The activated TF JUN also regulates the proliferation-associated gene* PSMD11* and the DNA repair-associated gene* RPS20*. There is evidence that curcumin (diferuloylmethane) can suppress tumor initiation, promotion, and metastasis. Curcumin can also inhibit the expression of JUN [43]. Additionally, the RNAi-induced induction of* ECT2* suppresses cell migration, invasion, and metastasis [44]. Our results indicate that the upregulation of* ECT2* in stage 4 bladder cancer cells is regulated by epigenetic regulation of* ECT2* expression. This is also supported by the significant change in the DNA methylation profiles in* ECT2* between normal bladder cells and stage 4 bladder cancer cells (*p* value < 0.007).

### 3.6. Functional Module Network Analysis in Bladder Carcinogenesis

The activated DNA repair of bladder cancer cells leads to metastasis owing to the immortality of cancer cells.

**Figure 5:**
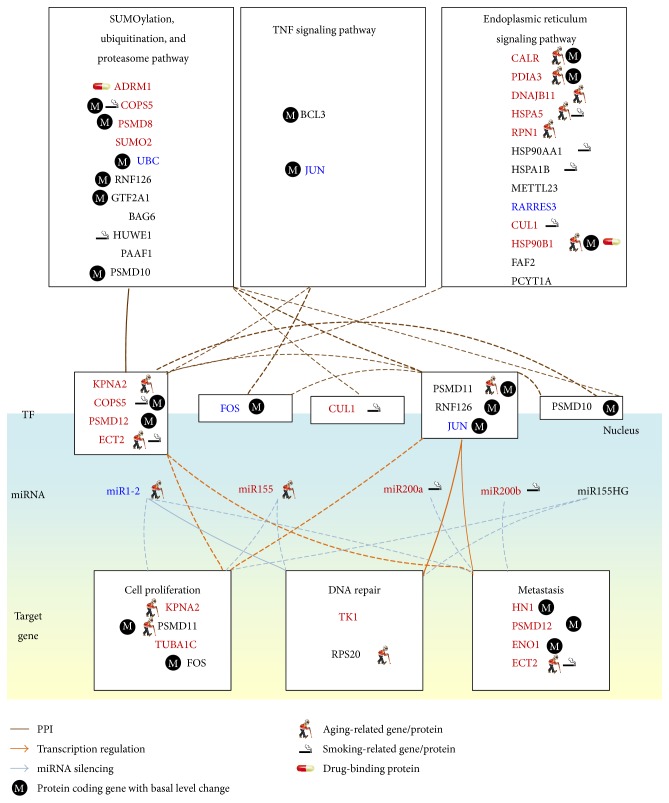
Module network of the core network biomarkers in Figure 3 for investigating the bladder carcinogenic mechanisms from normal bladder cells to stage 1 bladder cancer cells. The notations of gene/miRNA symbols and line styles are the same as those in Figure 3. The activated TFs KPNA2, COPS5, PSMD12, and ECT2 play an important role in mediating the signal transduction of the SUP and ER pathways to activate cell proliferation and metastasis in stage 1 bladder cancer. The metastasis of the stage 1 bladder cancer is repressed by the activated miRNAs miR200a and miR200b.

**Figure 6:**
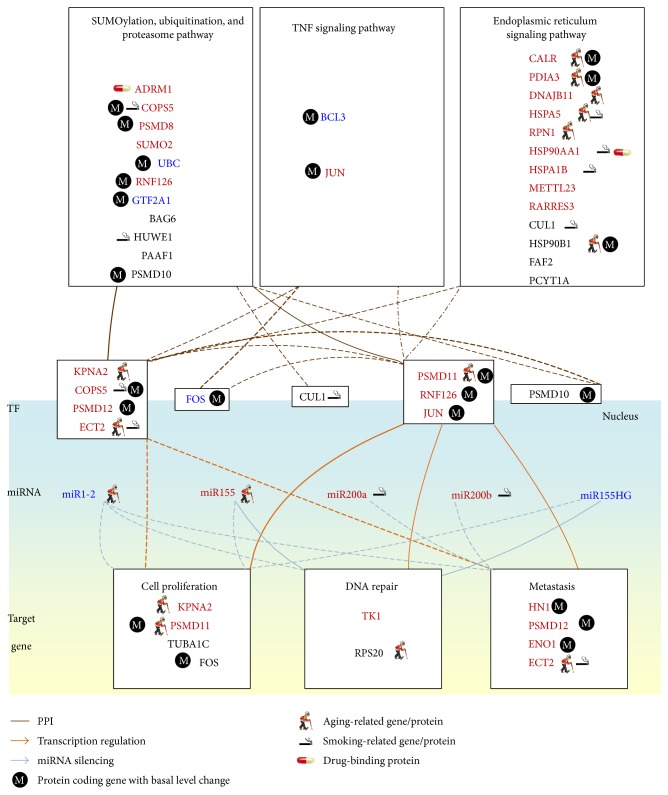
Module network of the core network biomarkers in Figure 4 to investigate the bladder carcinogenic mechanisms from stage 1 to stage 4 bladder cancer cells. The notations of gene/miRNA symbols and line styles are the same as those in Figure 4. The activated DNA repair of bladder cancer cells leads to metastasis owing to the immortality of cancer cells. The activated JUN in the TNF pathway induces cell proliferation, DNA repair, and metastasis in stage 4 bladder cancer cells.

According to the modular information in the GO database and the KEGG pathway database, the genes/proteins in the core network biomarkers (Figures 5 and 6) are projected into three pathways, the SUP pathway, the TNF signaling pathway, and the ER signaling pathway, and three biological processes: cell proliferation, DNA repair, and metastasis. The module networks in Figures 5 and 6 show that the activated TFs KPNA2, COPS5, PSMD12, and ECT2 play an important role in mediating the signal transduction of the SUP and ER pathways to activate cell proliferation and metastasis in stage 1 bladder cancer. The metastasis of the stage 1 bladder cancer is repressed by the activated miRNAs miR200a and miR200b, as shown in Figure 5. The activated signal transduction from SUP and ER pathways also triggers DNA repair through the epigenetically regulated TFs PSMD11 and RNF126.

Additionally, the activated TFs PSMD11, RNF126, and JUN mediate the signal transduction from SUP, TNF, and ER pathways to trigger cell proliferation, DNA repair, and metastasis in stage 4 bladder cancer, as shown in Figure 6. Although miR155 is activated in stage 1/4 bladder cancer, miR155 suppresses FOS and RPS20 in stage 1 bladder cancer, and miR155 only suppresses the DNA repair-associated gene* RPS20* in stage 4 bladder cancer. Furthermore, we suggest that DNA repair may play a critical role in repairing DNA damage, which results from genetic and epigenetic alterations, leading to phenotypic change of the bladder cells from normal cells to stage 1 cancer cells, and from stage 1 cancer cells to metastatic cancer cells.

**Figure 7:**
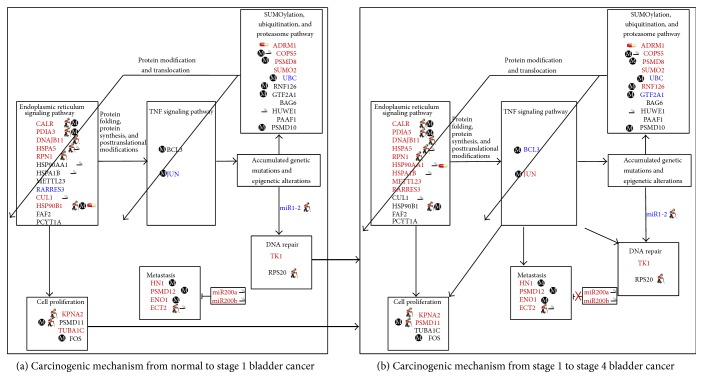
The carcinogenic mechanisms from normal to stage 1 bladder cancer cells (a), and from stage 1 to stage 4 bladder cancer cells (b). When the accumulated genetic mutations and epigenetic alterations lead to the dysregulation of the TNF pathway in inflammation, the accumulated misfolded proteins in the ER pathway induce cell proliferation in stage 1 bladder cancer (a). The regulations of ER and TNF pathways are adaptive to the accumulated genetic mutations and epigenetic alterations through the SUP pathway. The progression of DNA repair and cell proliferation in stage 1 bladder cancer ultimately results not only in the repression of miR200a and miR200b during metastasis, but also in the regulation of the TNF pathway to metastasis, cell proliferation, and DNA repair in stage 4 bladder cancer (b).

In summary, aging and epigenetic regulation dominate bladder carcinogenesis through CALR, PDIA3, DNAJB11, HSPA5, RPN1, HSP90B1, KPNA2, ECT2, and PSMD11 and through COPS5, PSMD8, RNF126, CALR, PDIA3, HSP90B1, PSMD12, PSMD11, JUN, HN1, and ENO1, respectively. Smoking promotes bladder carcinogenesis especially in metastasis. Finally, the cellular mechanisms from normal to stage 1 bladder cancer cells and from stage 1 to stage 4 bladder cancer cells are summarized in Figures 7(a) and 7(b), respectively. When the accumulated genetic mutations and epigenetic alterations lead to the dysregulation of the TNF pathway in inflammation, the accumulated misfolded proteins in the ER pathway induce cell proliferation in stage 1 bladder cancer (Figure 7(a)). Regulation of the ER and TNF pathways adapts to the accumulated genetic mutations and epigenetic alterations through the SUP pathway. The progression of DNA repair and cell proliferation in stage 1 bladder cancer ultimately results not only in the repression of miR200a and miR200b during metastasis, but also in the regulation of the TNF pathway to metastasis, cell proliferation, and DNA repair in stage 4 bladder cancer (Figure 7(b)).

### 3.7. Two Separate Drug Combinations for Treating Stage 1 and Stage 4 Bladder Cancer Cells with Minimal Side-Effects

The design of a multiple drug combination for treating stage 1 bladder cancer depends on a strategy of inhibiting the highly expressed genes* ADRM1*,* COPS5*,* PSMD8*,* SUMO2*,* CALR*,* PDIA3*,* DNAJB11*,* HSPA5*,* RPN1*,* CUL1*,* HSP90B1*,* KPNA2*,* PSMD12*,* ECT2*,* TK1*,* TUBA1C*,* HN1*, and* ENO1*; activating the suppressed genes* UBC*,* JUN*,* RARRES3*, and* FOS*; and suppressing the drug's effect on the nondifferentially expressed genes* BAG6*,* HUWE1*,* PAAF1*,* PSMD10*,* FAF2*,* PCYT1A*, and* PSMD10*. According to the drug design strategy (see Section 2), a multiple drug combination comprising gefitinib, estradiol, yohimbine, and fulvestrant was obtained for treating stage 1 bladder cancer.

The design of a multiple drug combination for treating stage 4 bladder cancer depends on a strategy of inhibiting the highly expressed genes* ADRM1*,* COPS5*,* PSMD8*,* SUMO2*,* RNF126*,* CALR*,* PDIA3*,* DNAJB11*,* HSPA5*,* RPN1*,* HSP90AA1*,* HSPA1B*,* METTL23*,* RARRES3*,* KPNA2*,* PSMD12*,* ECT2*,* JUN*,* TK1*,* TUBA1C*,* HN1*, and* ENO1*; activating the suppressed genes* BCL3*,* FOS*,* UBC*, and* GTF2A1*; and suppressing the drug's effect on the nondifferentially expressed genes, which are the same as those in stage 1 bladder cancer. We obtained a multiple drug combination comprising gefitinib, estradiol, chlorpromazine, and LY294002 for treating stage 4 bladder cancer. According to the information in DGIdb, miR-155, the HSP90 protein family, ADRM1, and estrogen receptor are the direct targets of the multiple drug combination comprising gefitinib, estradiol, yohimbine, and fulvestrant in stage 1 bladder cancer, respectively (Figures 3 and 5), while the same proteins are also the direct targets of the multiple drug combination comprising gefitinib, estradiol, chlorpromazine, and LY294002 in stage 4 bladder cancer, respectively (Figures 4 and 6). Moreover, the analysis of drug response genome-wide microarray data reveals that high doses of yohimbine can activate BAG6 in stage 1 bladder cancer, while high doses of chlorpromazine can activate HSPA5 and JUN in stage 4 bladder cancer. Therefore, low-dose yohimbine and low-dose chlorpromazine could avoid side-effects in the treatment of stage 1 and stage 4 bladder cancer cells, respectively. Ultimately, we designed one specific drug combination for treating stage 1 bladder cancer and another specific drug combination for treating stage 4 bladder cancer with minimal side-effects (Table 1).

## 4. Conclusion

In this study, we proposed a new method for constructing an IGEN for characterizing cellular mechanisms in bladder carcinogenesis by using system regression modeling and large-scale database mining. We then applied PGP to obtain the core network biomarkers of the IGEN. By comparing the connection changes of the core network biomarkers between normal bladder cells and stage 1 bladder cancer cells and between stage 1 and stage 4 bladder cancer cells, we investigated the progression mechanisms of bladder carcinogenesis. Database mining provided all possible candidates for genetic and miRNA regulations and protein interactions in IGEN. We used AIC and statistical assessment to prune the false positive regulations and interactions by applying the regression coupling model to NGS data and methylation profiles. We compared the connection differences in the core network biomarkers between different cellular types to explore bladder carcinogenic mechanisms. According to the comparison of the connection changes in the core network biomarkers between normal cells and stage 1 cancer cells and between stage 1 and stage 4 cancer cells, we investigated how the genetic and epigenetic regulations, miRNA regulations, and aging-related and smoking-related genes affect the biological functions that lead to bladder carcinogenesis. According to gene expression changes in the core network biomarkers between normal bladder cells and stage 1 bladder cancer cells and between stage 1 and stage 4 bladder cancer cells, we then identified two separate drug combinations for treating stage 1 and 4 bladder cancer cells. Therefore, the proposed IGEN construction method and PGP provide potential network biomarkers for bladder cancer diagnosis and treatment.

## Figures and Tables

**Table 1:** The multiple drug design strategy and potential multiple drug combination for stage 1 and 4 cancers.

	Stage 1 bladder cancer	Stage 4 bladder cancer
The highly expressed genes for potential inhibition strategy of multiple drug design	*ADRM1*, *COPS5*, *PSMD8*, *SUMO2*, *CALR*, *PDIA3*, *DNAJB11*, *HSPA5*, *RPN1*, *CUL1*, *HSP90B1*, *KPNA2*, *PSMD12*, *ECT2*, *TK1*, *TUBA1C*, *HN1*, and *ENO1*	*ADRM1*, *COPS5*, *PSMD8*, *SUMO2*, *RNF126*, *CALR*, *PDIA3*, *DNAJB11*, *HSPA5*, *RPN1*, *HSP90AA1*, *HSPA1B*, *METTL23*, *RARRES3*, *KPNA2*, *PSMD12*, *ECT2*, *JUN*, *TK1*, *TUBA1C*, *HN1*, and *ENO1*

The suppressed genes for potential activation strategy of multiple drug design	*UBC*, *JUN*, *RARRES3*, and *FOS*	*BCL3*, *FOS*, *UBC*, and *GTF2A1*

The nondifferentially expressed genes to avoid side-effect of multiple drug design	*BAG6*, *HUWE1*, *PAAF1*, *PSMD10*, *FAF2*, *PCYT1A*, and *PSMD10*	*BAG6*, *HUWE1*, *PAAF1*, *PSMD10*, *FAF2*, *PCYT1A*, and *PSMD10*

The potential multiple drug combination